# What motivates rural teachers to retain? A study on Chinese rural teachers' turnover from grounded theory and FsQCA

**DOI:** 10.3389/fpsyg.2022.998422

**Published:** 2023-01-30

**Authors:** Chao Cheng, Yanjie Diao, Xing Ding

**Affiliations:** Jing Hengyi School of Education, Hangzhou Normal University, Hangzhou, China

**Keywords:** rural teachers, retention intention, grounded theory, Fuzzy-set Qualitative Comparative Analysis (FsQCA), configurations

## Abstract

This research is aim at a deeper understanding of factors that leading Chinese rural teachers' (CRTs) turnover in their profession. The study regarded in-service CRTs (*n* = 408) as the participants, adopted the semi-structured interview and an online questionnaire to collect the data, and used grounded theory and FsQCA to analysis the data based. We have found that (A) welfare allowance, emotional support, and working environment can be substituted equivalently to increase CRTs' retention intention while professional identity regarded as the core condition; (B) career development is neglected for two reasons: one is CRTs compensated for inadequate social support by self-improvement; another is CRTs adapted themselves to accept professional stagnation; (C) the CRTs will have a strong intention to retain but lack enthusiasm for teaching because lack professional identity when the external environment is favorable. This study clarified the complicated causal relationships between CRTs' retention intention and its factors, contributed to the practical development of CRTs workforce.

## 1. Introduction

Teachers' turnover is a significant problem in educational research aspect, it refers either to area transfer within the teaching profession, such as changing the subject(s) to be taught, to migration from one school to another or to leaving the teaching profession (Boe et al., 2007). Scholar has pointed that teachers' turnover will cause some serious consequences for teaching quality (Hanushek, 2016), previous studies have found that teachers' turnover will cause a shortage of teachers and disrupt the regular teaching order (Loeb et al., 2005), as well as influence the coherence of teaching delivery, interpersonal relationships between teachers (Guin, 2014). Besides, teachers' turnover also affects educational equity because differential sorting of teachers across schools often results in inequitable distributions of teacher effectiveness (Nguyen et al., 2020). Moreover, several studies have highlighted that teachers' turnover will negatively affects students' achievements (Ronfeldt et al., 2013; Sorensen and Ladd, 2020) and further influences students' motivation (Guin, 2014). In terms of school, teachers' turnover increases school expenses (Ronfeldt et al., 2013; Nguyen et al., 2020; Sorensen and Ladd, 2020), impacts schools' effectiveness overall (Hong, 2010) and destroy the school's cultural legacy (Wang, 2014). In addition of these effects, teachers' turnover will continuously cause burnout and invisible turnover among teachers (Sorensen and Ladd, 2020).

### 1.1. Teachers' turnover: A worldwide problem

Data from different countries has pointed that teachers' turnover is becoming a worldwide problem from the past decades. For instance, teachers' turnover rate ranges from 13% to 15% annually in the USA (Ingersoll, 2002), based on the data, Boe et al. (2007) have pointed out that the turnover rate of teachers are still increasing. In Finland, where the teaching profession has been highly appreciated (Räsänen et al., 2020), but 40%−50% teachers had turnover intentions which remarkably persistent (Pyhältö et al., 2015; Räsänen et al., 2020) and 10%−15% of them were taken the turnover behavior (Nissinen and Välijärvi, 2011). In some development country such as Rwanda, 20% of teachers will leave the profession and 11% of them are from public-section teaching workforce (Zeitlin, 2020). Similarly, teachers' turnover also occurred in Australia and England (Hong, 2010).

Besides, some studies showed that the beginner teacher will leave the teaching profession in their early career period (Hughes, 2012; Redding and Henry, 2019), and previous study has corroborated this conclusion, around 33% of beginner teachers will leave the profession within 5 years after becoming the fully qualified teachers in Norwegian municipalities (Tiplic et al., 2015). Except the beginner teacher, there also some teachers (67–70 years) will choose to leave before they reach their maximum retirement age (Tiplic et al., 2015).

### 1.2. Teachers' turnover in Chinese rural context

Like other countries, statistical data from Liaoning province in China gave the evidence that teachers' turnover has consistently indicated a high rate (Wang, 2007). Therefore, teachers' turnover has become a growing concern in China especially in Chinese rural areas. Previous studies show that Chinese rural teachers (CRTs) have a weaken intention to retain in their profession (Lin et al., 2019) and 71.56% of them who want to turnover from rural schools to urban schools (Wang and Wu, 2019). Consequently, teachers' turnover characterized by an imbalance between Chinese rural and urban areas on the whole. In addition, a study from tracking survey of math teachers in northwest Chinese elementary schools revealed that teachers' turnover rate is 12.06% and 6.5% of them will be turnover to other professions (Chang et al., 2021), although previous studies have reported that Chinese government invested substantial recourses to recruit, develop, motivate, and retain effect rural teachers (Wei and Zhou, 2019).

Teachers provide the basis for the development of rural education, enhance the quality of rural education, and play a crucial role in promoting educational equity between urban and rural areas. Therefore, this research is aimed at a deep exploration on the influential factors of CRTs' retention intention and its complicated causal relationships. We expected to provide suggestions toward to reduce teachers' turnover in Chinese rural areas and address other concerns, such as rural teachers' occupational burnout. Moreover, this study also provides educational leaders worldwide with an example of the Chinese perspective on the matter. To do these, the following research questions were set out:

*RQ1: What factors have influenced Chinese rural teachers' turnover?*
*RQ2: How do the factors interactive combine to influence Chinese rural teachers' turnover?*

## 2. Literature review

### 2.1. Teachers' turnover factors

Factors are the significant research aspect to teachers' turnover. Currently, scholars have a comprehensive exploration on teachers' turnover factors and positively constructed the corresponding theoretical framework on it. For instance, Ingersoll (2001) analyzed the effects of three aspects of teacher characteristics, school characteristics, and organizational conditions through organizational analysis. Borman and Dowling (2008) conducted a meta-analysis on teachers' turnover, they identified five broad factors: teacher characteristics; teacher qualifications; school organizational characteristics; school resources; and student body characteristics. Nguyen and Springer (2021) organized the determinants of teachers' turnover into personal correlates, school correlates, and external correlates. Collectively, teachers' turnover factors can be separated in three dimensions: social, organizational, and individual factors.

#### 2.1.1. Social factors

In terms of social factors, it includes national and regional policy, teachers' salaries and benefits, and teacher professional development systems. Numerous studies have found that merit pay has a positive effect on teacher retention intention, and the salary increase will reduce the likelihood of teacher turnover (Rubenstein et al., 2017). As for policy factors, such as the Teacher Incentive Fund in the USA (Harrell et al., 2004) and the Supporting Plan for Rural Teachers and the Special Post Teachers program in China (Zhao, 2019), all have been indicated policy factors may influence the teacher labor market and teacher retention decisions.

#### 2.1.2. Organizational factors

Regarding organizational factors, such as vocational training (Kizilaslan, 2012), collaborative school culture, and professional development barriers (Van den Borre et al., 2021) are crucial for rural teachers with their turnover problems. Teachers with heavy workload show a high intention to turnover. Besides, teachers who are less likely to leave in schools with better working conditions, which are characterized by better locations, better facilities, teaching assignments that better match teachers' expertise, and fewer student discipline problems (Nguyen et al., 2020). Moreover, diversity climate was examined that contributed to turnover intention (Seriwatana, 2021).

#### 2.1.3. Individual factors

The individual factors included teachers' characteristics, qualifications, and reactions to jobs. Previous studies have explored teachers' turnover individual factors such as teachers' age, gender, educational level, and teaching experience. For instance, some scholars have reported that male, young and novice teachers have a higher turnover rate than others (Conley and You, 2009; Ingersoll and May, 2012). In addition, teachers' reactions to their jobs have noteworthy impact on turnover intention. Teachers' professional identity, motivation (Grant et al., 2019), job satisfaction, self-efficacy (Troesch and Bauer, 2020), and work engagement (Tvedt et al., 2019) were negatively related to teachers' turnover intention, while burnout showed a positive relationship with teachers' intentions to quit (Madigan and Kim, 2021).

### 2.2. Relationship between teachers' turnover and its factors

Literature above demonstrated studies that conducted in the same aspect such as teacher or school factors. However, it lacks a holistic perspective. As the study progressed, scholars have realized that teachers' turnover was affected by several equally important factors rather than a single individual or contextual one (Clandinin et al., 2014). As a result, researchers have begun to focus on the impact of the combination effect of factors on teachers' turnover and explore the relationships between the factors. These studies have exhibited in two research progressions.

One is the quantitative study in which variables are introduced to analyze the pattern of influence transmission between factors. Several studies focused on job satisfaction and burnout, which are considered to be important predictors of teachers' turnover. For instance, belonging and emotional exhaustion are key variables in mediating the impact of school context variables on job satisfaction and motivation to turnover (Skaalvik and Skaalvik, 2011). And time pressure and discipline problems enhanced the influence of burnout on teachers' turnover (Li et al., 2020). In addition, findings from Kalyanamitra et al. (2020) demonstrated that Training Facilities, Benefits and Compensation, and Performance Appraisal will impact the retention intention through the development of skills and increased satisfaction. Jarinto et al. (2019) have reported the mediating role of employee commitment toward the organization as a mediator for employees in Thailand higher education institutions.

Another type of research attempts to analyze teachers' turnover theoretically. Simultaneously, scholars pay attention to the different dimensions that influenced teachers' turnover. They have found, the interaction between the individual and the environment will influence teachers' turnover. For instance, rational choice theory (Kukla-Acevedo, 2009) discusses the relationship between teachers' interest demand and organizational resources; planned behavior theory pay attention to teachers' beliefs influenced by school environment (Kersaint et al., 2007); and personal-environmental fit theory from the perspective of individual and job fit (Vagi and Pivovarova, 2017). This is an attempt to adopt a correlational perspective analysis that incorporates teachers into educational organizations and educational phenomena into social systems.

### 2.3. The current study

Based on the literature review, the causal relationships between teachers' turnover and its factors present symmetrical and linear unidirectional, and the correlations between factors are independent. Although scholars have paid attention to the causal relationship between teachers' turnover and its factors. However, as a matter of fact, teachers' turnover and its factors are multiple and concurrent. Simultaneously, the factors are interdependent and interrelated, so that the causal relationship between teachers' turnover and its factors is complicated and asymmetrical. Unfortunately, there are no relevant studies have been discovered to clarify the complicated causal mechanisms between teachers' turnover and its factors, furthermore, previous studies have paid less attention to CRTs. Therefore, the current is aimed to explore the complicated causal relationship between CRTs' turnover and its factors. The study was conducted with CRTs and adopted a combined qualitative and quantitative method, to discuss and respond the causal complexity between CRTs' turnover and its factors based on the constructed theoretical framework of factors of CRTs' turnover.

## 3. Methodology

### 3.1. Participants

In this study, participants were in-service teachers who work in Chinese rural elementary schools, where the term rural teachers refer to teachers in impoverished towns and rural areas and they separated in two sets.

The one set of participants (*participants' demographic information is shown in* Table 1) from three rural elementary schools. Following the non-purpose sampling, 13 CRTs were invited randomly from three elementary schools to participate this study during the fieldwork period. At the beginning of this study, we select GT school, a rural elementary school in Zhejiang province as the first research target, we got the connection with GT school due to the first author's internship program. The identity of internship teacher helped the researcher got the admission into research filed and do the interview (Strauss and Corbin, 1990). Then, refer to the principle of snowball sampling, the first author invited internship supervisor work as the intermediary to make a connection with other two rural elementary schools.

**Table 1:** Participants' demographic information.

**Numbery**	**Gender**	**Age (years)**	**Education**	**Title**	**Length of services (years)**
X_1_-WT	Male	37	Bachelor	Primary	4
Y_1_-SYX	Female	42	Tertiary	Associate senior	15
X_2_-ZW	Male	31	Master	Middle	5
Y${}_{2}^{*}$-YR	Female	26	Bachelor	Primary	2
Y_3_-YJ	Female	30	Master	Primary	3
X_3_-XW	Male	35	Tertiary	Middle	9
Y_4_-BJ	Female	38	Bachelor	Middle	14
Y_5_-TJ	Female	25	Bachelor	Primary	2
Y_6_-TFF	Female	42	Bachelor	Associate senior	16
Y_7_-JY	Female	24	Bachelor	Primary	1
X_4_-YYF	Male	47	Bachelor	Senior	22
Y_8_-DD	Female	31	Master	Middle	6
X_5_-YXH	Male	36	Tertiary	Middle	12

Y${}_{2}^{*}$-YR is a particular case: after graduating, she began working at a primary school in a city. Then, after working in the city for two years, she worked in a rural school.

The other set of participants (*statistical participants' demographic information, see* Table 2) were from a 2-year rural teacher professional development program which is dominated by the third author, and this program is aimed to improve rural teachers' profession. Data from Chinese Ministry of Education has given the evidence that in the CRTs workforce, the number of female teachers is significantly higher than male teachers (Chinese Ministry of Education, 2020). Therefore, in this set of participants, the proportion of male CRTs is less than female CRTs. This set of participants are from the rural elementary schools in Zhejiang province, Shandong province, Jiangsu province, Beijing municipality, and Hunan province of China. Considering the program could intervened to the results, this study was conducted before the program started.

**Table 2:** Statistical participants' demographic information.

		**Number**	**Proportion (%)**			**Number**	**Proportion (%)**
Gender	Male	48	11.76	Title	Senior	2	0.49
	Female	360	88.23		Associate Senior	20	4.90
Ages (years)	Under 25 years	36	8.82		Middle	170	41.67
	25–35 years	256	62.75		Primary	216	52.94
	35–45 years	112	27.45	Length of services (years)	Under 1 year	27	6.86
	Over 45 years	40	9.80		1–2 years	40	9.80
	Master	16	3.92		2–3 years	28	6.86
Education	Bachelor	384	94.12		3–5 years	36	8.82
	Tertiary	8	1.96		Over 5 years	276	67.65

### 3.2. Data collection

In term of data collection, we adopted semi-structured interview to collect the qualitative data, and an online questionnaire to collect quantitative data.

The qualitative data were interview notes and recorded audios. We adopted the semi-structured interview to learn the experience and perception of Chinese teachers work in rural areas, the questions were added or omitted depending on the way of discussion is development.

The quantitative data was responded questionnaires from a rural teachers' professional development program. The dimensions of the questionnaire were referred to the result of qualitative data. To facilitated distribution and statistic, we utilized the online questionnaire to learn the CRTs' retention intentions and its factors. The questionnaire was regarded the influential factors as the antecedent condition, and CRTs' retention intentions as the outcome condition, and was developed that used the five-point Likert scale with the 1–5 anchors.

### 3.3. Data analysis

The two categories of data collected in this study were mainly analyzed using the grounded theory (Strauss and Corbin, 1990) to deal with the qualitative data, and the Fuzzy-set Qualitative Comparative Analysis (FsQCA) (Ragin, 1987) was adopted to analysis the quantitative data.

To answer the research question 1, the grounded theory was employed to construct the factors of CRTs' turnover theoretical framework. In this part, the interview notes and audios were be given the priority to analysis. The grounded theory is aimed to construct a bottom-up theoretical framework based on fieldwork (Chen, 2000) that could realistically identify the real needs of CRTs. Additionally, the constructed theoretical framework provided the theoretical dimensions for the application of the FsQCA.

To answer the research question 2, we employed the FsQCA to explore the complicated causal relationships between CRTs' turnover and its factors. The FsQCA is primarily used to analyze complicated causal relationships between configurations and out comes with cases (Du and Jia, 2017). In this part, the responded questionnaires were the main data, the interview notes and audios were regarded as the supplementary material for analysis. The FsQCA provides two configurations: core and peripheral conditions, which could be marked as present, absent, or “blank.” Core conditions are components with a strong causal relationship, while peripheral conditions are components with a wear causal relationship (Fiss, 2011). These configurations could provide a comprehensive explanation of the complicated causal relationships between CRTs' turnover and its factors, as well as trigger further discussion.

### 3.4. Research ethic

Before the interview, the researcher disclosed himself to participants of basic personal information, explained the purpose and content of this study, and obtained the authorization of record. All participant's personal information and collected data were anonymized in this study. In addition of this, the Review Board at the Jing Hengyi School of Education and Academic Ethical Committee of Hangzhou Normal University had provided all ethical approval before performing the research.

## 4. Findings

### 4.1. Result from grounded theory

**Figure 1:**
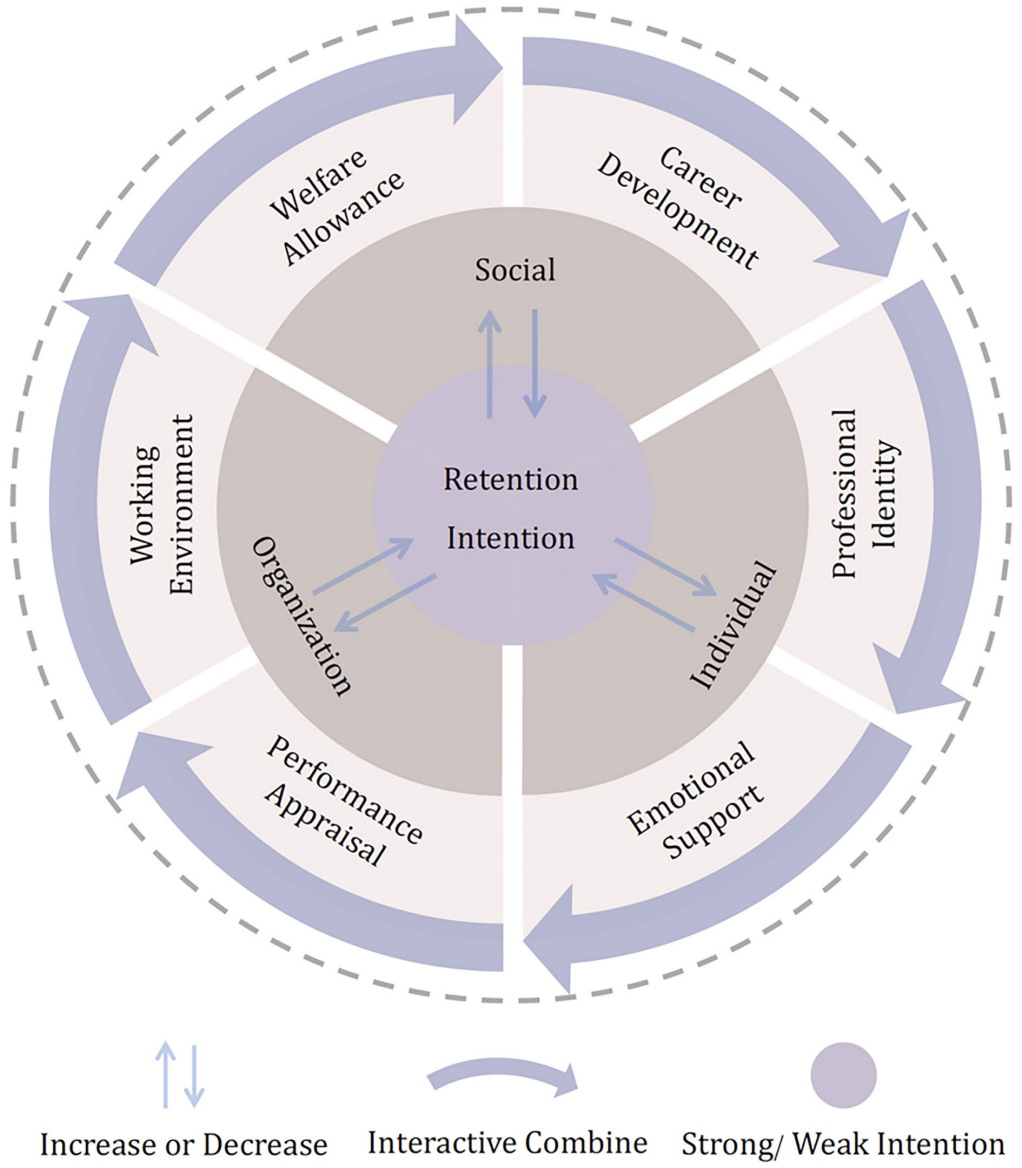
Factors of CRTs' turnover theoretical framework.

In this study, we used the qualitative software Nvivo 12 Plus to assist categorized and coding the participants' answers based on grounded theory. After open coding, axial coding, and select coding, we have constructed the factors of CRTs' turnover theoretical framework (depicted in Figure 1). This constructed theoretical framework indicates that the factors of CRTs' turnover come from in three dimensions: Social factors (SF), Organization factors (OF), Individual factors (IF). Including Welfare allowance (WA), Career development (CD), Working environment (WE), Performance appraisal (PA), Emotional support (ES), and Professional identity (PI) six factors. These factors will increase or decrease CRTs retention intention. Moreover, the combination of these factors will increase or decrease CRTs' retention intention.

### 4.2. Measurements

Firstly, 70 questionnaires were pre-distributed and collected at random to check the scientific validity of the questionnaire before the formal survey. Then, we adopted the quantitative research software SPSS 26.0 to test the reliability, validity, and correlation of the questionnaire. Next, we have totally distributed 440 questionnaires, 32 invalid questionnaires were eliminated due to the quality of the responded answers, and it remained 408 valid questionnaires.

#### 4.2.1. Testing for reliability and validity

This study utilized Cronbach's α and CR (Construct Reliability) to test the questionnaire's reliability. As shown in Table 3, the Cronbach's α and CR for each variable on the questionnaire were >0.7, indicating that the questionnaire had excellent internal consistency. The structural validity determined by factor analysis showed that the overall validity KMO (*Kaiser-Meyer-Olkin*) value was 0.901 (*sig*. = *0.000*), indicating that the questionnaire had excellent general validity. Moreover, the AVE (*Average Variance Extracted*) values for each variable were >0.5, showing that the questionnaire indicated good discriminant validity.

**Table 3:** Testing for reliability and validity.

	**Measurement contents**	**KMO**	**Cronbach's α**	**AVE**	**CR**
Social factors	Welfare allowance		0.883	0.669	0.857
	Career development		0.753	0.616	0.758
Organizational factors	Working environment	0.901	0.818	0.601	0.817
	Performance appraisal		0.831	0.682	0.811
Individual factors	Emotional support		0.874	0.676	0.861
	Professional identity		0.858	0.560	0.791

#### 4.2.2. Testing for correlation

Table 4 has demonstrated the testing for correlating antecedent conditions and outcome condition of the CRTs' turnover and its factors. According to the correlation test's two-tailed results, the *p*-values of each antecedent condition and the outcome condition are < 0.01, indicating a significant correlation between the antecedent conditions and the outcome conditions, which is consistent with the expected results.

**Table 4:** Testing for correlation.

	**Retention Intention**	**Welfare allowance**	**Career Development**	**Working environment**	**Performance Appraisal**	**Emotional Support**	**Professional identity**
Retention intention	1						
Welfare allowance	0.561	1					
Career development	0.352	0.664	1				
Working environment	0.464	0.721	0.606	1			
Performance appraisal	0.411	0.609	0.681	0.578	1		
Emotional support	0.510	0.667	0.664	0.651	0.698	1	
Professional identity	0.492	0.461	0.565	0.528	0.514	0.709	1

### 4.3. Results from FsQCA

In this study, we used the software FsQCA 3.0 to calibrate the data, identify configurations, and construct configurations, based on 408 sample from the questionnaire.

#### 4.3.1. Data calibration

The FsQCA requires the original data to be calibrated to a range of values between 0 and 1. Specifically, 1 denotes complete affiliation, 0 denotes complete disaffiliation, and the range between 0 and 1 is continuous. In addition, the FsQCA converts each variable into a calibration set by setting three meaningful anchor points: complete affiliation, intersection, and complete disaffiliation. The calibration could indicate whether the variable is included or excluded from the set (Ragin, 1987). Referred to Fiss (2011), set three meaningful anchor points on the Likert 5-point scale: 5 represents complete affiliation, 3 represents intersection, and 1 represents complete disaffiliation.

#### 4.3.2. Configurations identification

The FsQCA clarifies the necessity and sufficiency of combining each antecedent condition for the outcome by determining whether a subset relationship exists between the antecedent condition set (X_i_) and the outcome condition set (Y_i_), with the obtained configurations serving as the core for identification (Ragin, 1987). Consistency [*Consistency* (*Y*_*i*_ ≤ *X*_*i*_) = Σ*min* (*X*_*i*_*, Y*_*i*_)*/*Σ(*Y*_*i*_)] is the degree to which cases correspond to the set-theoretic relationships expressed in a configuration (Fiss, 2011), whereas coverage [*Coverage* (*Y*_*i*_ ≤ *X*_*i*_) = Σ*min* (*X*_*i*_*, Y*_*i*_)*/*Σ(*X*_*i*_)] evaluates the empirical significance of a consistent subset (Ragin, 1987). To calibrate the data, the necessary conditions (see Table 5) were needed. In general, a consistency of >0.90 for the antecedent condition is regarded as the necessary condition for the outcome (Ragin, 1987).

**Table 5:** Necessary conditions analysis of factors influencing the intention to stay in education.

**Antecedent conditions**		**Strong intention**		**Weak intention**	
		**Consistency**	**Coverage**	**Consistency**	**Coverage**
Social factors	Welfare allowance	0.847	0.912	0.810	0.484
	~Welfare allowance	0.520	0.831	0.852	0.756
	Career development	0.810	0.867	0.839	0.491
	~Career development	0.519	0.853	0.755	0.689
	Working environment	0.796	0.880	0.786	0.481
Organizational factors	~Working environment	0.530	0.817	0.802	0.686
	Performance appraisal	0.878	0.835	0.867	0.457
	~Performance appraisal	0.429	0.853	0.687	0.758
	Emotional support	0.908	0.831	0.886	0.500
Individual factors	~Emotional support	0.399	0.863	0.667	0.801
	Professional identity	0.944	0.805	0.587	0.430
	~Professional identity	0.332	0.868	0.909	0.852

We set CRTs' retention intention as the outcome condition, and the factors as antecedent conditions. The result has reported that the consistency of the antecedent conditions “ES,” “PI,” and “~PI” are >0.9, indicating that “ES” and “PI” were the core conditions for CRTs' strong intention to remain. “~PI” was the necessary condition for the rural teachers' weak intention to retain their profession.

#### 4.3.3. Configurations construction

According to Ragin's recommendation, case frequency >1, consistency higher than 0.9 and PRI consistency >0.75 were set as screening thresholds to construct truth tables (Ragin, 1987). In this study, the condition of simultaneous occurrence of intermediate and parsimonious solutions was adopted as the core condition, and the configurations state was presented in the form of intermediate and parsimonious solution fits. According to this, we determined four configurations of strong intention and three configurations of weak intention (see Table 6). The consistency of configurations is >0.8, and the consistency of configurations is >0.9, implying that Configurations L_1_, L_2_, L_3_, and L_4_ constitute sufficient conditions for CRTs with a strong intention, whilst Configurations R_1_, R_2_, and R_3_ constitute sufficient conditions for CRTs with a weak intention.

**Table 6:** Configurations of rural teachers' retention intention influential factors.

**Antecedent conditions**	**Strong intention**				**Weak intention**		
	**L**_1_	**L**_2_	**L**_3_	**L**_4_	**R**_1_	**R**_2_	**R**_3_
Welfare allowance	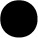	°	°	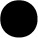	°	○	○
Career development	○	°	°	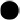	○	○	○
Working environment	○	○	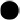	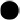	○	○	°
Performance appraisal	○	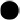	○	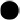	○	°	○
Emotional support	○	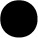	○	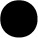	○	○	○
Professional identity	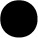	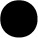	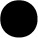		○	○	○
Consistency	0.890	0.921	0.930	0.947	0.940	0.950	0.954
Raw coverage	0.323	0.412	0.312	0.708	0.457	0.437	0.438
Unique coverage	0.033	0.033	0.014	0.002	0.031	0.014	0.013
Solution consistency	0.900				0.932		
Solution coverage	0.827				0.483		

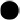
 indicate the presence of peripheral conditions, 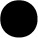 indicate the presence of core conditions, ° indicate the absence of peripheral conditions, and ^○^ indicate the absence of core conditions. “Blank” indicate “Don't Care”.

In detail, there are two driving paths of configurations that generate a strong intention to retain among CRTs. (A) The driving paths of a single IF as the core condition. Configuration L_2_ (~WA^*^ ~WE^*^ PA^*^ ES^*^ *PI*) and Configuration L_3_ (~WA^*^ WE^*^ ~PA^*^ ~ES^*^ *PI*). The results demonstrated that a positive PI^*^ WE or good PI^*^ ES^*^ PA will motivate CRTs to retain in their profession. However, single IF cannot achieve this result and should be supplemented by good OFs. (2) The driving path of dual SF and IF as core conditions. Configuration L_1_ (WA^*^ ~CD^*^ ~WE^*^ ~PA^*^ ~ES^*^ *PI*) and Configuration L_4_ (WA^*^ CD^*^ WE^*^ PA^*^ *ES*). The results demonstrated that reasonable WA^*^ *PI* or good WA^*^ CD^*^ WE^*^ PA^*^ *ES* will elicit strong intention among CRTs. This implies that, in addition to the condition of high WA, a single condition of strong PI or excellent ES, together with good CD or WE or PA, will motivate CRTs to have a strong intention. In addition, there are three configurations R_1_, R_2_, and R_3_ that will trigger the weak intention of CRTs. Limited CD, ES, and PI are core conditions, whereas disparities in WA, PE, and WE are peripheral conditions.

## 5. Discussions

### 5.1. Indispensable social support

When factor PI regard as the core condition, but the presence of PI alone cannot retain CRTs; other conditions should complement it. Comparing configurations L_1_, L_2_, and L_3_ reveals that when rural teachers have a high PI, conditions WA, ES, and PA, and WE show a potential substitution relationship. According to social support theory, the subjects of support can be divided into formal social networks such as government and school, and informal social networks such as family, neighbors, and colleagues; the support content can be divided into instrumental support and emotional support (Thoits, 1982). Three configurations differ in the subject and specific social support content. WA and WE can be considered instrumental support provided by formal social networks, and ES can be regarded as emotional social support supplied by informal social networks.

Configuration L_1_ reported that PI and WA as core conditions, CRTs are eager to retain proving that salaries and welfare are incentives for teachers, but they are not the only incentives. In configuration L_3_, the WE indicates that rural teachers have convenient transportation, high-quality educational facilities, and living conditions. According to Maslow's hierarchy of needs theory, the WE satisfies the basic physiological and security needs of rural teachers. It empowers a sense of work-life balance, which has also been proven to be a crucial incentive (Robbins, 2008). Although inadequate financial rewards might weaken teachers' PI, emotional rewards in configuration L_2_ can emerge as a compensatory measure, and rich emotional rewards can also strengthen teachers' PI (Xiong and Gao, 2020). When teachers are satisfied with the social support, they will identify more with the responsibility and value of joining rural education, and they will develop a strong sense of professional mission and the intention to maintain (Li, 2021). In general, those with a strong sense of professional mission are less concerned with monetary compensation. They emphasize the self-actualization and social significance of their careers more (Wrzesniewski et al., 1997), which explains why teachers in Configuration L_2_ regard PA as a peripheral condition.

### 5.2. Neglected career development

Limited CD opportunity is the factor in teachers' turnover (Jin et al., 2021). According to recent studies, rural teachers face a dual dilemma of insufficient post-employment training and difficulty in title progression (Chen and Li, 2021). In this study, CD is a peripheral condition in Configuration L_4_, absent in Configuration L_1_, and the “blank” in Configurations L_2_ and L_3_. This implies that CD is not a core condition to increasing rural teachers' intention to retain. Then, why do rural teachers with poor career development conditions have such a strong intention to remain in their positions?

The personal-environmental fit theory of career development sheds lights on our analysis of rural teachers' retention: CD is the result of the interplay of various matching relationships between individuals and the WE, and the better the individual fits with the WE and the more the environment can meet the individual's career expectations, the stronger the individual's intention to retain (Kristof-Brown et al., 2005). Two orientations characterized the interaction between the person and the environment: alteration of the environment and adaptation of the environment. As a result of the environment's modification, rural teachers can make full use of the available resources to enable their CD expectations. Therefore, when teachers have a strong sense of PI and mission, they can pursue CD opportunities *via* their own efforts, even if the formal social network offers less support for teachers. Adaptation of the environment suggests that environmental conditions become a significant component in shaping teachers' behavior and that teachers can adapt their expectations to the environment. The fieldwork also provides a realistic depiction of how rural teachers adjust to their environment: some rural teachers accept the stagnation of their career development amid environmental constraints. “*...... Associate Senior is difficult to evaluate, I have participated in the selection process for several years but always failed, the middle tittle is enough in the rural areas, I think”* (*2022-5-6, X*_2_*-ZW*). The identity of official staff has been less attractive to younger generations in recent years, although it continues to be an important factor in teachers' career decisions (Wei and Zhou, 2019). Teacher Y_2_-YR worked in a private school in a city on the eastern coast after she graduate. She stated, “*My family thinks it's a wonderful idea for girls to be the official staff at elementary schools, since it guarantees you'll never be jobless”* (*2022-5-9, Y*_2_*-YR*). Urban schools have better prospects, but private school means unofficial staff identity, so Teacher Y_2_-YR abandoned her urban employment and reapplied to rural schools in her hometown for a position with official staff. Based on the follow-up interview, Teacher Y_2_-YR thought, “*I don't want to achieve anything after became an official staff in my school, completing daily teaching duties is enough”* (*2022-5-9, Y*_2_*-YR*). Obtaining the opportunities as a member of the official staff has satisfied the needs of rural teachers. Consequently, they gradually lose the expectation of CD. Consideration should be given about whether rural education should afford the price of low quality when rural teachers have voluntarily neglected their career development.

### 5.3. Does retention imply the ability to teach well?

Comparing Configurations L_1_, L_2_, L_3_, and L_4_, Configuration L_4_ lacks the core condition of PI to other configurations. However, it creates an excellent job for rural teachers with excellent WA^*^ comfortable WE^*^ reasonable PA^*^ ES. These favorable conditions satisfy rural teachers with the external environment, and they have a strong intention to retain despite the absence of the core condition of PI. Teacher X_1_-WT corroborates Configuration L_4_ for us by stating, “*I enjoy teaching in a GT school, and I find satisfaction when I work here”* (*2022-4-27, X*_1_*-WT*). GT school, a rural elementary school in Zhejiang province of China, where Teacher X_1_-WT works at. To attract the rural teachers to retain, GT school has built a new dormitory and provides three meals per day for teachers. In addition, due to the remote location of GT School, “*we get an extra allowance than other rural teachers in XS County, and the welfare from the trade union in festivals is also good” (2022-4-27, X*_1_*-WT)*, therefore Teacher X_1_-WT is relatively satisfied with his income and the welfare. In addition, teacher X_1_-WT had access to several resources and opportunities despite working in a rural school, “*……the experts from H University helped me develop the school-based curriculum, I learned a great deal in the process” (2022-4-27, X*_1_*-WT)*. The process of school-based curriculum development has assisted Teacher X_1_-WT in his personal growth. Arguably, the circumstance of teacher X_1_-WT could mirror the truth of Configuration L_4_.

A further point to consider is that although the consistency of Configuration L_4_ is the highest among the four strong intentions configurations, it claimed that Configuration L_4_ would most stimulate rural teachers' retention intention. However, does strong retention intention means strong work enthusiasm? From the observation of Teacher X_1_-WT in daily teaching activities, the researcher noted that “*Teacher X*_1_*-WT frequently smokes in the corridor. In the meantime, the students are watching movies in the class”* (*2022-4-27, Researcher*), based on Teacher X1-WT's daily teaching activities observation. Because of the lack of PI, Teacher X_1_-WT was always perfunctory in his work and gradually developed burnout as a rural teacher, “*Actually, there are no school requirements for me, the only request is that I complete daily teaching activities. Anyway, I am the official staff of GT School, the school has no authority to remove me”* (*2022-4-27, X*_1_*-WT*). Therefore, it is obvious that rural teachers who have a strong retention intention do not always have a strong work enthusiasm.

### 5.4. Expand discussion: comparisons on urban and rural teachers' turnover

We have discussed four configurations that enhance teachers' retention intention to decrease CRTs' turnover, while CD, ES, and PI appear as core conditions in each of the configurations in the analysis of what leads to weak retention intention. This suggests that the absence of these three predicted the likelihood of teachers' turnover. The result is in line with earlier study, according to Li et al. (2020), who showed that the primary causes of urban teachers' turnover were a lack of favorable DF prospects, an absence of a PI, and a low social standing. This demonstrates that the desire for CD is a key factor in both urban and rural teacher movement. Being an unofficial employee left many teachers, particularly in the urban private teacher groups, lack opportunities for title progression and CD. This uncertain professional life increased teachers' anxiety and intention to turnover to public schools (Gu and Bi, 2021). China has adopted the “county management and school employment” teacher exchange and rotation policy to ensure the balanced development of compulsory education. The policy had ruled that public school teachers have to turnover between the schools in their county, and “rural school teaching experience” has become a requirement for teachers to receive senior titles. To further their careers, urban teachers are now becoming more mobile and teaching in rural areas as a result of policy needs.

Salary may the essential factor influencing teachers' turnover and the reason why many urban public-school teachers give up their staffing to join private schools (Borman and Dowling, 2008; Gu and Bi, 2021). However, in this study, it was not a core condition that led to the weak retention of rural teachers, that is, it was not a determinant factor in rural teachers' turnover. The reason for this may be that with the implementation of the “Supporting Plan for Rural Teachers,” teachers in some rural areas were able to receive government subsidies, which improved their WA in some extent (Zhao, 2019). Some previous studies have also confirmed that subsidies in rural areas can reduce teacher turnover, and that long-term subsidies are more effective than one-time subsidies (Clotfelter et al., 2008).

## 6. Conclusion

In this study, we employed grounded theory to construct factors of CRTs' turnover theoretical framework. Then, used FsQCA to generate configurations and discuss the driving paths based on 408 CRTs. This study has found: (A) four configurations were formed to constitute the strong intention of CRTs to retain, which was mainly divided into two paths: one is the driving path of individual single-factor as the core condition, including the Configuration L_2_ with reasonable PA, good ES and strong PI, and the Configuration L_3_ with the excellent WE and good PI. Another is the driving path of social and individual dual-factors as the core conditions, including the Configuration L_1_ with a high WA and a positive PI. Configuration L_4_ indicates a generous WA, excellent CD, reasonable PA, and good ES. (B) The potential substitution of SF, OF, IF suggests that when PI becomes a core condition, conditions of WA, ES, and We could be substituted equivalently to increase CRTs' retention intention in the same way. In addition, CD does not exist as the core condition to influence CRTs' retention intention, possibly for two reasons: first, CRTs improve themselves to compensate for inadequate social support, and second, CRTs adapt themselves to accept professional stagnation. (C) Improving WA, investing in ES, and fostering a sense PI are all feasible strategies to decrease CRTs turnover.

The implication of this study is to make a practical contribution to reducing teacher turnover, promoting the development of the rural teaching force, and improving the quality of rural education. We employed the FsQCA to discuss the complicated causal relationship between CRTs' turnover and its factors, which include, social, organizational, and personal factors.

Firstly, the study demonstrated that when teachers are more satisfied with social support, especially emotional support, they are more likely to stay in rural areas. A study of teacher retention in rural schools in Ghana gave the evidence that both structural capital and social capital are critical for retention of teachers in rural schools (Opoku et al., 2019). Local education authorities should give rural teachers the necessary respect and care for their wellbeing. In addition, schools can increase interaction between teachers and locals and promote cultural adaptation of rural teachers by organizing local cultural events and student service-learning activities in local communities (Schafft, 2016). Similarly, teachers need to work in a school atmosphere of unity and mutual support, studies such as the one by Helms-Lorenz et al. (2015) further suggest that schools can reduce attrition by providing coaching and support, by engaging teachers in teams or networks and as such prevent isolation.

Secondly, there is a link between employment practices and teachers' retention intentions. Stability makes teachers more likely to stay in their jobs, which was also verified in a study of rural teachers in Australia, show that contractual employment disrupts the development of a sense of belonging to the profession and the building of meaningful connections between teachers and their schools (Plunkett and Dyson, 2011).

Finally, this study also found that simply increasing teachers' retention intentions does not achieve the goal of improving rural teaching quality. Lack of enthusiasm for teaching and professional identity can make it difficult for teachers to teach well even when they have a good working environment, and even rural teachers may actively adapt to the poorer career development system in rural schools. To increase teacher retention, it is crucial to give rural teachers a better career path and to foster their sense of professional identity. According to several studies, giving teachers more autonomy over their teaching might boost their intentions to stay (Grant et al., 2020). Many countries are beginning to explore ways to promote teacher professional development by giving teachers greater autonomy over their teaching and enhancing teaching resources. For instance, “the Framework curriculum” in Finland was seen to provide teachers a great deal of freedom to select and/or interpret the subject matter as well as the opportunity to experiment with different teaching strategies (Webb et al., 2004). Results from the Dutch context also appear to suggest that teacher education institutions, in addition to schools, should play a significant role in reducing teacher turnover (den Brok et al., 2017). These cases have revealed that attrition is less likely when teachers are well-prepared throughout teacher education.

As most empirical research, this study has limitations. Firstly, this study's sample was collected from CRTs, so that the findings may not be applied directly in other countries, but they can serve as a useful reference. Moreover, the distribution of educational resources in China's rural eastern and western regions is unequal (Bao, 2020), Therefore, it is debatable if it is reasonable to set the anchor based on Likert 5-point scale, even though the data sampled rural areas in multiple provinces. In addition, Following Ragin's recommendation, the FsQCA is appropriate for small and medium-sized samples analysis (Ragin, 1987), so whether the configurations in this study can adequately explain the group dynamics of teachers' turnover need to be further discussed.

## Data availability statement

The original contributions presented in the study are included in the article/supplementary material, further inquiries can be directed to the corresponding authors.

## Ethics statement

Written informed consent was obtained from the individual(s) for the publication of any potentially identifiable images or data included in this article.

## Author contributions

CC: introduction, methodology, findings, conclusion, data collection, and data analysis. YD: discussion, literature review, and data collection. XD: data collection. All authors contributed to the article and approved the submitted version.
